# Shock and awe or incentive-compatible harm reduction? Graphic health warnings on tobacco packages

**DOI:** 10.1186/s12954-021-00487-3

**Published:** 2021-04-16

**Authors:** Ian Irvine, Hai V. Nguyen

**Affiliations:** 1 Department of Economics, Concordia University, S-H 1155-11, Henry F. Hall Building, 1455 De Maisonneuve Blvd. W, Montreal, Canada; 2 School of Pharmacy, Memorial University of Newfoundland, St. John’s, Canada

**Keywords:** Food and drug administration, Graphic health warnings, Smoking, Tobacco, Canada, US

## Abstract

**Background:**

Graphic Health Warnings (GHWs) on cigarette packages were first introduced in Canada in 2001 and will become mandatory in the US as of January 2022. While previous studies have evaluated the impacts of GHWs, the data used in these studies have several shortcomings. The objective of this paper was to investigate the likely impact of such warnings in the US based upon the experience of Canada using hitherto unexplored monthly cigarette sales data, and to explore if alternative approaches involving risk-reduced products might be more successful in reducing smoking.

**Methods:**

We used quasi-experimental segmented regression and difference-in-differences analyses. Data on monthly sales (i.e., shipments) of cigarettes from Canadian manufacturers to Canadian retailers during 1995–2005 were obtained from Statistics Canada.

**Results:**

We found that GHWs did not have a significant impact on the sales of cigarettes in Canada. We propose an alternative type of graphical health messaging that actively combines information on how to quit with the legally required messaging. The novelty of the proposal is that it is incentive compatible for the supply side of the market and if adopted in several states, the measure could be tested by using a suitable treatment–control design.

**Conclusions:**

Our findings imply that we should not expect any notable decline in sales or consumption as a result of implementation of GHWs in the US. The main impact of GHWs will be to add to the anti-smoking culture that has grown steadily over several decades, and this may impact smoking in the longer term.

## Introduction

The World Health Organization's Framework Convention on Tobacco Control requires its members to use Graphic Health Warnings (GHWs) on tobacco packaging. The health policy community views them as an essential strategy to reduce tobacco use. Canada was the first country to introduce GHWs, in January of 2001. The US Food and Drug Administration (FDA), with the power given to it under the Tobacco Control Act (TCA), recently required that such warnings be put in place on US cigarette packages.^1^ In response, the tobacco industry has filed lawsuits seeking to invalidate this policy [2].

The purpose of this paper is to investigate the likely impact of such warnings in the US based upon the experiences of Canada, and to explore if alternative approaches involving risk-reduced products (RRPs) might be more successful in reducing smoking. The FDA proposed graphic warnings as long ago as 2009; they were intended to replace the existing text-only warnings. However, that initiative was overturned in court, upon appeal by the tobacco manufacturers. The Court ruled in August 2012, following an injunction in November 2011, that the FDA’s graphic warning requirements violated the tobacco companies’ First Amendment rights and the FDA had not provided sufficient evidence to demonstrate the efficacy of the GHWs they were proposing [3].

The evidence originally proposed by the FDA was based upon a comparison of Canadian and US smoking prevalence patterns surrounding the time when GHWs were introduced in Canada. The Court deemed the evidence to be too weak to warrant implementing GHWs. However, some recent papers, which we review below, have claimed that the introduction of such warnings in Canada did indeed reduce smoking prevalence—in one case by as much as 19%.^2^ We illustrate that these claims are not tenable.

Claims of substantial smoking prevalence reduction following GHWs suggest that there remains low-hanging fruit yet unpicked in the area of tobacco-control policy. But caution is in order. For example, the introduction of plain packaging in the Australia tobacco market in 2012 had minor impacts on smoking. As described by the Australian Government Department of Health, plain packaging, combined with several other measures including large tax-induced price increases, reduced sales minimally [8].^3^

Another factor that casts doubt on a large GHW effect in Canada is the coincidence of the implementation of GHWs with a sizeable and prolonged increase in taxes and prices. Between December 2000 and December 2003, the national aggregate real price index for cigarettes in Canada rose by almost 70%. For the years 2004 and 2005 the real price index was essentially stable. A standard price elasticity of demand for cigarettes estimate from the literature for the period around 2001 falls in the neighborhood of − 0.3, though more recent estimates using store-level data suggest an estimate in the neighborhood of − 0.1 [12, 13]. Using, conservatively, an estimate of − 0.2 suggests that quantity consumed might have decreased by 14% during this 3-year period following the price increase of 70%.

The policy part of the paper involves an exploration of whether there exist more effective harm-reduction messaging policies in the current era. The nicotine marketplace in 2020 bears very little resemblance to its form in 2001. A host of RRPs, that are also termed alternative nicotine delivery systems (ANDs), are now available that were not available then, and these products have impacted cigarette sales in both the US and Canada^4^. We illustrate that positive ‘switching’ messaging (i.e., messaging that nudges smokers to transition to RRPs) on cigarette packages are incentive compatible for producers and may be more effective in reducing smoking than GHWs purely of the shock-and-awe type that the FDA has commissioned.

This paper first reviews earlier research. We then identify data sources that can be used in the analysis. We then detail the econometric modeling using Canada and US monthly data at the time GHW's were introduced in Canada. The results are then presented. In the final sections we propose an alternative program of action that could be implemented to reduce smoking and improve health.

## Background

The theory underlying the introduction of GHWs has two threads. In the first instance, warnings can constitute a source of information on the negative and potentially deadly outcomes that are associated with smoking. But surveys indicate that few smokers are unaware of adverse outcomes. Hence the potential for GHWs to impact smoking prevalence and quantity smoked comes primarily from ideas in behavioral economics. Bernheim and Rangell [14] and Gul and Pessendorfer [15] propose that individual decisions are frequently based on cues or temptation. In their world, graphic health warnings are a counter cue, or a countervailing message to the temptation to smoke. GHWs may transfer individuals who have been hit with a smoking cue into a ‘cold’ state from a ‘hot’ state. But the ultimate value of such behavioral nudges must be empirically validated.

The first paper to evaluate the Canadian GHWs econometrically was Gospodinov and Irvine [16]. Their analysis used individual-level data from the Canadian Tobacco Use Monitoring Surveys (CTUMS), merged with price data by province and month. They did not detect any impact of the introduction of the warning labels on smoking prevalence and found just some mild evidence favoring an impact on daily consumption. The period covering their analysis, however, was relatively short, comparing smoking behavior six months prior to the introduction of the warnings with smoking behavior in the six months following their introduction.^5^

More recent studies used data for a longer time period to evaluate the policy. Azagha and Sharaf [18] used 1998–2008 panel data from the National Population Health Survey (NPHS). They concluded that the odds of smoking declined by 13% as a result of the introduction of GHWs. The FDA analysis [19] used annual data on smoking rates and covariates for the period 1991–2009 for both Canada and the US. The FDA researchers estimated a regression for Canada and the US separately for the years prior to the introduction of the GHWs in Canada. They then projected the regression relationship for the post-GHWs period and computed a mean difference between the predicted smoking rates with the actual smoking rates. The difference for Canada was substantial, but the difference was also substantial for the US. In other words, the behavior of the treatment group was not very different from the behavior of the control group. Hence, the FDA could not conclude that the GHWs impacted smoking rates substantially.

Huang et al. [20] used essentially the same data as the FDA but estimated a difference-in-differences (DD) model using both the US and Canadian data in a single regression. This has the advantage of having somewhat greater statistical power: rather than having four separate groups of observations, the DD approach conserves on degrees of freedom by imposing common response patterns on some of the parameters of the model. They found a large, negative and statistically significant policy coefficient. This is the basis for their strong conclusion on the effectiveness of the GHWs, namely, the GHWs reduced smoking prevalence by between 12% and 19%.

The data used in these three studies, however, have shortcomings. While Azagba and Sharaf's paper brings longitudinal data to bear on the problem, even these data are not ideal: the surveys run on a 2-year cycle, with interviews in the months May, July and September of even years (here 2000) and January of the odd year (here 2001). ‘Dropped’ cases are chased up in April of the uneven years. Given the sampling timetable, there appear to be no observations between end-January 2001 and May 2002, with the exception of some ‘dropped’ interviews. De facto, their specifications all seem to amount to turning on the dummy in May of 2002—16 months after the introduction of the policy. They do not report estimates based on a January 2001 switch-on date for the GHW dummy—the date of their legal introduction.

The annual data used in both the FDA and the Huang et al. studies come from several different sources—four for the Canadian data and two for the US data. The Canadian data come from the General Social Survey, the Survey of Smoking in Canada, the NPHS, and the CTUMS [21]. These surveys, however, are not all implemented in the same manner despite a similarity in the questions asked. Some are by telephone only, some are by telephone and in-person, and the NPHS interview method varies with the year in which the survey is implemented. Gilmore [22], in his detailed survey of Canadian data sources, discusses the similarities and dissimilarities of the questions posed to survey respondents, and indicates that in some cases responses can be validly compared across surveys and in other cases not.

Furthermore, surveys that focus solely upon tobacco use may yield different reported smoking rates than general health surveys that contain a module on smoking. For example, the Canadian Community Health Surveys (CCHS), which are repeated cross-section general health surveys, similar in focus to the longitudinal NPHS, report consistently higher smoking prevalence rates than the CTUMS, which focus only upon smoking. Table 1 indicates that, in addition to higher average rates through time in the CCHS data, there is substantial variation in the degree of understatement in the CTUMS data. In particular, between 2001 and 2003, the smoking prevalence rate reported by the CTUMS data declines by 0.8 points, whereas in the CCHS data it declines by 2.9 points—almost a four-fold difference. Between 2003 and 2005, the decline in the CTUMS smoking prevalence rate is almost twice the decline in the CCHS. From that point in time onwards, the differences are less dramatic. But the very large discrepancies between 2001 and 2005 suggest that alternative or additional data should be explored. The CCHS series may be more reliable on account of its greater number of interviews (135,000 vs 20,000) in these years. The critical year 2001 is the year with the biggest difference between the CCHS and CTUMS smoking prevalence rates. The average difference for years excluding 2001 is 2.9 points but the CTUMS rate for 2001 is 4.3 points below the CCHS rate. These large differences indicate that additional sources of data need to be explored.^6^

**Table 1:** Smoking Prevalence in Canada (% of population), CTUMS versus CCHS. *Source*: Health Canada, CTUMS, and CCHS (see references)

	CTUMS			CCHS	(CTUMS–CCHS)
	Total	Daily	Occasional	Total	
1999	25.2	20.9	4.3		
2000	24.4	19.9	4.7		
2001	21.7	18.1	3.7	26.0	− 4.3
2002	21.4	17.6	3.9		
2003	20.9	16.6	4.2	23.1	− 2.2
2004	19.6	15.0	4.6		
2005	18.7	15.0	3.7	21.8	− 3.1
2006	18.6	14.4	4.3		
2007	19.2	15.3	3.9	22.1	− 2.9
2008	17.9	13.5	4.4	21.5	− 3.6
2009	17.5	13.6	4.0	20.2	− 2.7

## Monthly data on sales, smoking prevalence and production

In order to avoid the pitfall of multiple data sources, our primary data series is the monthly quantity of sales (i.e., shipments) from Canadian manufacturers to Canadian retailers (CANSIM table 303-0062 and 303-0007). Month-to-month sales data from retailers to smokers do not exist. Sales predate consumption: Manufacturers maintain about one month of sales in the form of inventory (inventory data come from the same CANSIM table). Annual sales, as described in Table 2, declined by 1.7% between 2000 and 2001, and by 14.6% between 2001 and 2003. This pattern differs strongly from the CTUMS pattern, which sees smoking prevalence decline substantially in 2001, and by very little in the following two years. It is worth repeating that real prices increased by 70% in this short period. The US has similar monthly data on sales [23]. As with the Canadian data, US inventories equal about one month of such sales.

**Table 2:** Annual sales and production of cigarettes, Canada 1998–2003. *Source*: CANSIM 303-0007

	Sales in millions	Sales %Δ	Production in millions	% of annual production in Nov and Dec
1998	45,579		48,730	16.4
1999	45,112	− 1.0	47,224	16.0
2000	43,033	− 4.6	46,068	15.8
2001	42,295	− 1.7	44,403	15.5
2002	38,155	− 7.9	41,267	14.7
2003	36,099	− 5.4	37,929	17.2

We generated a monthly series on smoking prevalence based upon the CTUMS. There is no need to use the CTUMS data in annual form; the online CTUMS database at the Statistics Canada website can be quizzed to yield a monthly smoking prevalence series, to which we marry a matching monthly price series. It is surprising that there exist no econometric models to date that exploit the monthly availability of the sales data and the CTUMS smoking prevalence data. The use of monthly data from the CTUMS alone provides additional degrees of freedom before and after the GHW policy.

We focus on sales data for the period of 1995–2005 to avoid measurement error associated with smuggling activity and illegal sales activity. A well-documented smuggling period came to an abrupt end at the second quarter of 1994 because of excise tax reductions by the Canadian federal government, which were matched by most provinces—tobacco is a shared tax jurisdiction in Canada.^7^ The post-2005 period is ruled out because police reports indicate that the market for illegal tobacco began to grow again about this time, reaching its peak in the years 2007–2009.

In our robustness checks, we use Canadian monthly production data and US monthly sales data to examine specific aspects of the estimation. The production data enable us to examine the possible impact of stocking by producers prior to the GHW event.

## Econometric analysis of GHWs in Canada

### Segmented regression

An ‘event’ such as the introduction of GHWs can influence smoking immediately, or may require time to impact smokers, or with both an immediate and a lagged effect. An immediate impact might be anticipated given that a large percentage of smokers claim that they are interested in quitting: the GHWs might act as a tipping mechanism. In contrast, addiction is difficult to overcome and GHWs may both prompt smokers to make more frequent quit attempts (a small proportion of which may ultimately result in success), and reinforce an anti-smoking culture.

We analyze the potential for both an immediate and lagged impact using a segmented or piecewise regression model, also termed ‘Interrupted Time Series’ analysis (ITS) [25]. In this segmented regression, an immediate impact can be modelled with a dummy shift in the relationship where the dummy is switched on at the time of the event. The lagged effect can be modelled as a change in the slope of a time trend. Hence, the specification of this regression model is:

1 $$Y_{t} = \beta_{0} + \beta_{1} Trend + \beta_{2} PolicyPeriod_{t} + \beta_{3} PolicyPeriod_{t} \times PostpolicyTrend_{t} + \beta_{4} X_{t} + \varepsilon_{t} ,$$

where *Y*_*t*_ are outcomes (sales, smoking prevalence or production, in log) in month *t*; *Trend* is a linear time trend; *PolicyPeriod* is a dummy variable equal to 1 if the policy is in effect in month *t*, and equal to 0 otherwise; *PolicyPeriod* × *PostpolicyTrend* is the interaction term between *PolicyPeriod* and the post-policy time trend. *β*_*2*_ and *β*_3_ are the main parameters of interest: *β*_2_ estimates the change in the level of the trend post-policy, while *β*_3_ estimates the change in its slope. *X* is a vector of aggregate controls including cigarette prices (lagged one month), alcohol prices, and seasonally adjusted unemployment rates. *ε*_*t*_ is the error term. All data are in monthly form.

### Difference-in-differences regression

A threat to the segmented model is that an intervention or a shock happening in Canada at the same time as the GHW may confound the estimated effect of the policy. If that is the case, it is not possible to separate the effect of the policy from that of confounding factors. To address this potential confounding issue, we conduct a DD analysis that uses the US as a control country, following the Huang et al. study. The US data are fruitful because their price pattern differs from the price pattern experienced in Canada. The cultural similarities between Canada and the US, combined with similar economic cycles, mean that the US data form a meaningful control group.

This type of analysis [26–28] computes the changes in the outcomes in Canada following the introduction of the GHW policy, and compares those with corresponding changes estimated for the US. Our main DD regression model uses monthly country-level sales data from Canada and the US for the same study period, i.e., 1995–2005, and takes the form:

2 $$Y_{t} = \beta_{0} + \beta_{1} Canada + \beta_{2} (Canada \times PolicyPeriod_{t} ) + \beta_{3} X_{t} + D_{t} + \varepsilon_{t} ,$$

where *Y*_*t*_ are the outcomes in month *t*, and the right-hand-side covariates are as described above. *Canada* is an indicator variable equal to one for Canada, the treatment country, and zero for the US, the control country. $D_{t}$ is a vector of year dummies which account for secular and seasonal changes or shocks in outcomes that are common to the treatment and control groups.

The coefficient of interest is *β*_*2*_ on the interaction term between the *Canada* indicator and the *PolicyPeriod* indicator. This coefficient captures the effects of the GHW policy on outcomes for Canada relative to the associated change in outcomes for the US. For ease of interpretation of the marginal effects, all the regressions are estimated using the linear probability model [29]. Robust standard errors are reported.

We note that there is no difference in the ‘dosage’ of the policy measure being considered here. Powell, Pacula, and Jacobson [30] and Courtemanche et al. [31] analyze federal policies that have differential impacts depending on the state implementing them. No individual province has the authority to dilute or enhance the graphic messaging in Canada, and no individual state in the US has that authority either.

## Results

### Descriptive analysis

**Fig. 1:**
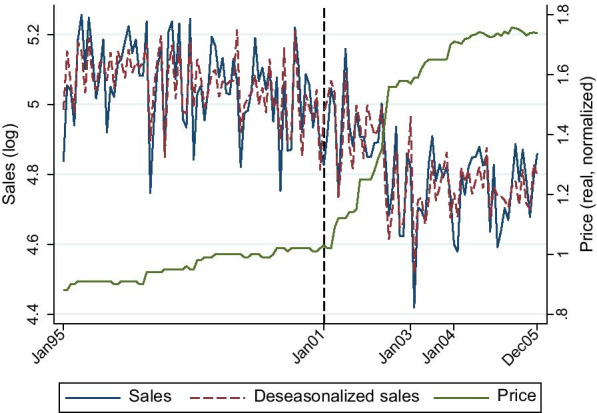
Canada monthly per capita sales (in log) and prices, 1995–2005.* Sources*: Canada’s monthly cigarette sales are from CANSIM Tables 303-0062 and 303-0007. Canada’s monthly cigarette prices are from CANSIM 326-0020

**Fig. 2:**
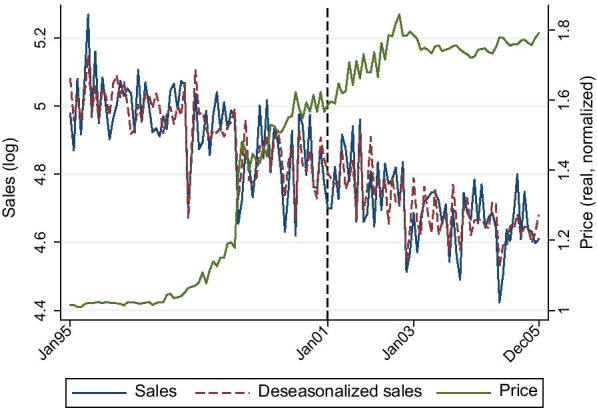
US monthly per capita sales (in log) and prices, 1995–2005. *Sources*: US monthly cigarette sales are from https://www.ttb.gov/tobacco/tobacco-stats.shtml. Prices are from Federal Reserve Bank of St Louis: CWSR0000SEGA, Consumer Price Index for Urban Wage Earners and Clerical Workers: Tobacco and smoking products, Index 1982–1984 = 100, Monthly, seasonally adjusted

**Fig. 3:**
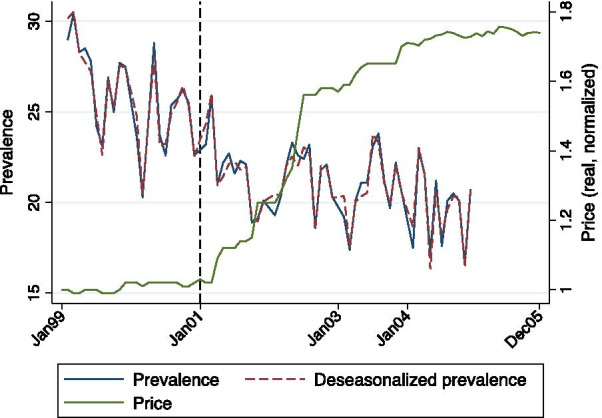
Canada monthly smoking prevalence and prices, 1999–2005. *Sources*: Canada monthly smoking prevalence of smoking data are extracted from the Canadian Tobacco Use Monitoring Surveys (CTUMS), using the ODESI access system (Ontario Data Documentation Extraction Service Infrastructure). Canada monthly cigarette prices are from CANSIM 326-0020

Figures 1, 2 and 3 present monthly Canadian sales, US sales, and Canadian smoking prevalence, respectively, matched by corresponding Canadian and US monthly real normalized price data for the period 1995–2005. Sales are per capita and in log form and smoking prevalence is the percentage of the population that smoked (either daily or occasionally) in the preceding 30 days. The series are de-seasonalized using the ratio-to-moving-average method. The vertical dashed line in each figure indicates the date when the intervention came into effect, i.e., January 2001.

In Fig. 1, the pre-policy trend in Canadian sales appears negative, while the post-policy trend cannot be characterized so simply. The trend is similar to the pre-policy trend for almost two years post January 2001, and then the level of the series appears to drop. This pattern does not suggest an obvious policy impact. Further, the introduction of the GHWs in Canada coincided with the start of a very substantial rise in real prices. In Fig. 2, the US sales appear to decline faster than in Canada pre-GHW. This may reflect stronger US price increases that occurred prior to 2001. In fact, as shown later, once we control for prices, there is no difference in the sales trends between the US and Canada before the GHW introduction. Figure 3 shows that pre-policy smoking prevalence in Canada trends downward and that trend appears to continue through until 2002. At that point the series is not easily interpretable: smoking prevalence rises substantially during the months June–September 2002. We shall see from the estimation that the smoking prevalence pattern is not well-explained by any of the regression models.

### Regression results

#### Segmented regression—Canadian sales

Table 3 reports the regression estimates for the Canadian sales and smoking prevalence series. For each outcome, there are three columns of estimates. The first column contains a basic specification, where the dependent variable is a function of a constant, a trend, the price of retail cigarettes and additional controls. The second column reports the results after a dummy is included to capture a potential shift in the level of the outcome. The third column allows for both a shift in the level and a change in the slope of the relationship; it is the segmented regression specification.

**Table 3:** Segmented regression estimates, sales and smoking prevalence outcomes, Canada

	Canada sales			Canada smoking prevalence		
	(1)	(2)	(3)	(4)	(5)	(6)
Cigarette price	− 0.436***	− 0.453***	− 0.530***	6.985**	4.276	1.868
	(0.109)	(0.111)	(0.099)	(3.087)	(3.490)	(4.964)
Time trend	− 0.009***	− 0.007***	− 0.004	− 0.393***	− 0.263*	− 0.205
	(0.003)	(0.003)	(0.004)	(0.119)	(0.143)	(0.169)
GHW effect (level change)		− 0.051	− 0.045		− 1.708	− 0.824
		(0.039)	(0.031)		(1.108)	(1.615)
GHW effect (slope change)			0.002			0.127
			(0.002)			(0.124)
Unemployment rate	− 0.016	− 0.000	0.007	− 0.726	0.167	− 0.012
	(0.023)	(0.025)	(0.020)	(0.699)	(0.887)	(1.077)
Alcohol price	0.024***	0.023***	0.012	0.466	0.307	− 0.033
	(0.009)	(0.009)	(0.012)	(0.294)	(0.307)	(0.430)
Constant	1.492	1.435	3.421	− 41.787	− 22.719	32.680
	(1.434)	(1.419)	(2.098)	(48.496)	(49.371)	(81.225)
R^2^	0.73	0.73	0.84	0.64	0.65	0.65
N	131	131	131	66	66	66

Marginal effects from segmented regressions are reported. Columns 1–3 are based on deseasonalized monthly per capita sales data (in log) for the period of 1995–2005. Columns 4–6 are based on deseasonalized monthly smoking prevalence data for the period of 1999–2004. Robust standard errors are reported

*p < 0.1; **p < 0.05; ***p < 0.01

For the sales outcome, column 1 indicates that the time trend and price coefficients are each significant. The price elasticity of demand at the beginning of the period, when the normalized price level takes a value of 1, is − 0.436, and at the end of the period, when the price variable takes a value of 1.9, is − 0.828. The trend variable is significant at the 1% level of significance. In column 2, while small and negative, a dummy included for a potential shift in the level is insignificant. The price elasticity and time trend retain their significance; price elasticity changes very slightly in value, from − 0.436 to − 0.453 at the start of the period. In column 3, after including both a shift in the level and a change in the slope of the relationship, the price elasticity increases further to − 0.530, the time trend loses its significance and is smaller in magnitude. The level coefficient is negative but small and not statistically significant, indicating that we cannot reject the null hypothesis of no impact of the policy on the level of the outcomes. The slope coefficient is also insignificant and has a perverse sign –the trend is positive rather than negative. Together, these results confirm expectations based on visualizing the data presented in Fig. 1, namely, the raw data do not suggest a break in either the level or trend in this series at the time the GHWs were introduced.

#### Segmented regression—smoking prevalence outcome

The last three columns of Table 3 contain coefficient estimates for the smoking prevalence regressions. In contrast to the sales estimates, none of the specifications contains a credible combination of estimates. In the base specification, the price coefficient is significant but of the wrong sign, while the time trend is unusually strong. The coefficient estimate of − 0.393 implies a trend decline of above one third of a percentage point per month. Adding dummies for the level in column 5 and for both the level and the slope in column 6 does not lead to more credible estimates, and increases the explained sum of squares only marginally. The price coefficient stays positive and the trend coefficient remains large. To get a sense of how theoretically ‘wrong’ these coefficients are, note that the trend variable alone would reduce smoking prevalence from 25% to 0% within a decade. The regression says that decreases in prices prevented this from happening, because the price variable has a positive impact. We are forced to conclude that the use of higher frequency monthly data for smoking prevalence does not overcome the problems with the econometric estimates produced by earlier researchers who sacrificed degrees of freedom by using annual data from the CTUMS.

The regression estimates may be indicating that the CTUMS smoking prevalence data in the GHWs period are subject to considerable randomness. We noted above that the difference between the CTUMS and CCHS unconditional estimates was unusually large at the time the GHWs were introduced, in addition to the fact that the recorded decline in CTUMS smoking prevalence data was not mirrored in a decline in sales. We have examined the data from the CTUMS on the number of cigarettes smoked per day by continuing smokers, but we cannot detect any increase in this variable that would align the smoking prevalence data with the sales data.

#### Difference-in-differences regression—Canadian and US sales

We first examine if the time trends in sales for Canada and the US prior to the introduction of the GHWs are similar, an underlying assumption of the DD analysis. The results in column 1 of Table 4 indicate that, after controlling for prices in the regression, there is no significant difference in these pre-policy trends between Canada and the US. The results from the DD regressions without (with) a Canada-specific time trend are given in column 2 (column 3). The coefficients on the DD term are negative but small and not statistically significant. The Canada indicator is positive but not significant, indicating no difference in sales per capita between Canada and the US. The price coefficient is − 0.15 and statistically significant. Thus, the result of the DD analysis is consistent with that of the segmented regression analysis, namely, the null hypothesis of no GHW impact cannot be rejected.

**Table 4:** Difference-in-differences regression estimates, sales outcome, Canada and US

	Parallel trend assumption testing	DD analysis	
	(1)	(2)	(3)
GHW effect		− 0.038	− 0.057
		(0.037)	(0.045)
Canada dummy	0.177	0.156	0.089
	(0.240)	(0.132)	(0.154)
Canada specific time trend	0.001		0.001
	(0.001)		(0.001)
Cigarette price	0.003	− 0.148**	− 0.150**
	(0.118)	(0.058)	(0.059)
Unemployment rate	− 0.005	− 0.013	− 0.001
	(0.033)	(0.017)	(0.022)
Alcohol price	− 0.004	− 0.001	− 0.001
	(0.007)	(0.005)	(0.005)
*R*^2^	0.57	0.78	0.78
*N*	142	262	262

Analyses use deseasonalized monthly per capita sales data (in log) from Canada (treatment) and the US (control). The regression for pre-policy trend comparison uses 1995–2000 data. The difference-in-differences regression uses 1995–2005 data. Year fixed effects are included in regressions. Robust standard errors are reported

*p < 0.1; **p < 0.05; ***p < 0.01

### Robustness checks

#### Analysis of Canadian cigarette production data

It is possible that producers may have printed a disproportionately large run of cigarette packs bearing the pre-GHW labels prior to January 2001, leading to a false null GHW finding. To test this hypothesis formally, we estimated a regression of the log of production on a set of monthly dummies and a time trend. This regression produced predicted values of the dependent variable for each month. The predicted values for end-of-year 2000 should be significantly less than the actual values if producers did in fact produce an unusually high level of cigarettes bearing the ‘old’ label.

The regression indicated that the November actual production value was less than the prediction and the December actual value was greater than the prediction. These negative and positive residuals almost cancelled each other. In terms of the standard error of the dependent variable, the November residual was less than one standard error and the December residual was slightly greater than one standard error of the dependent variable. We also ran higher order polynomials in the trend, but the result was unchanged. This indicates that producers did not game the system by producing additional ‘old-label’ packs prior to January 2001.^8^

As another robustness check, we estimated a segmented model for the production outcome, i.e., with estimates for both the changes in level and slope. The estimates (column 1 of Table 5) indicate that while the price coefficient is negative and significant, the changes in level and slope are all insignificant. This suggests that the GHW policy has no effect on the level or the growth rate of production over time.

**Table 5:** Robustness checks, segmented regression

	Canada Production	Canada Sales, Tax	Canada Smoking prevalence, Tax	US sales	Dynamic effects, Canada Sales	Dynamic effects, Canada Prevalence	Canada sales, 1999–2004	Canada sales, 1999–2004	Canada sales, 1999–2004
	(1)	(2)	(3)	(4)	(5)	(6)	(7)	(8)	(9)
GHW effect (level)	− 0.037	− 0.048	− 0.823	0.011				− 0.030	− 0.009
	(0.046)	(0.030)	(1.618)	(0.018)				(0.062)	(0.053)
GHW effect (Slope)	0.000	0.002	0.133	0.000					0.007
	(0.003)	(0.002)	(0.117)	(0.001)					(0.005)
GHW effect (level, 1 year post)					− 0.018	0.553			
					(0.046)	(1.547)			
GHW effect (Level, 2 years post)					0.077	3.502*			
					(0.062)	(1.860)			
GHW effect (Level, 3 years post)					0.086	− 0.106			
					(0.054)	(2.139)			
GHW effect (Slope, 1 year post)					− 0.004	− 0.631*			
					(0.008)	(0.320)			
GHW effect (Slope, 2 years post)					− 0.012	0.518			
					(0.011)	(0.378)			
GHW effect (Slope, 3 years post)					0.018	0.208			
					(0.012)	(0.426)			
Cigarette tax		− 0.311***	0.935						
		(0.054)	(2.500)						
Cigarette price	− 0.356**			− 0.091*	− 0.264	6.720	− 0.435***	− 0.484***	− 0.663***
	(0.148)			(0.049)	(0.305)	(10.808)	(0.138)	(0.174)	(0.181)
Unemployment rate	− 0.009	0.006	0.020	− 0.009	0.005	1.897	− 0.037	− 0.021	− 0.012
	(0.030)	(0.020)	(1.045)	(0.007)	(0.023)	(1.306)	(0.031)	(0.040)	(0.038)
Alcohol price	0.020	0.011	− 0.040	0.002	0.007	− 0.273	0.040***	0.037***	0.021
	(0.016)	(0.012)	(0.426)	(0.004)	(0.013)	(0.465)	(0.013)	(0.014)	(0.019)
*R*^2^	0.68	0.85	0.65	0.94	0.85	0.69	0.60	0.60	0.81
*N*	131	131	66	191	131	66	71	71	71

##### Use of cigarette taxes instead of cigarette prices

The estimates based on cigarette taxes^9^ in place of cigarette prices are reported in columns 2–3 of Table 5. The coefficients are negative for the level effect and positive for the slope effect but not statistically significant for either sales or smoking prevalence outcomes. These results indicate that we cannot reject the null hypothesis of a zero impact of the GHWs.

##### Placebo test

We re-estimated the segmented regression with US sales as an outcome. As the US did not adopt GHWs, we expect the Canadian GHW policy has no impact on the US sales. The estimates (column 4) confirm this. Further, the US price is significant and negative, as expected. This exercise further confirms the validity of our use of the US as a control group in a difference-in-differences analysis.

##### Dynamic effects of GHWs

The analyses thus far estimate the average effect of the policy over the entire period after the policy intervention, i.e., February 2001 to December 2005. It is possible that the policy had an effect during some sub-periods and no effect in others, but overall, had an insignificant effect when averaging over the entire period. To address this possibility, we break the post-policy period into several sub-periods and examine whether there are effects in any of these. For the interrupted time series analysis, we experiment by turning on the level and slope variables at 12-month intervals moving forward in time. The results (columns 5–6) indicate no effect for GHWs in years 1, 2 and 3–5 after the introduction of GHWs.

##### Shorter time period

The regressions for the sales outcome in the above analyses used a longer time period (i.e., 1995–2005) compared with regressions for the smoking prevalence outcome (1999–2004). To enable comparison with the smoking prevalence outcome, we re-estimated the regressions for the sales outcome for the shorter duration, namely 1999–2004 (columns 7–9, Table 5). The results mirrored those for the longer duration.

##### Another test for pre-policy parallel trend assumption underlying difference-in-differences analysis

**Fig. 4:**
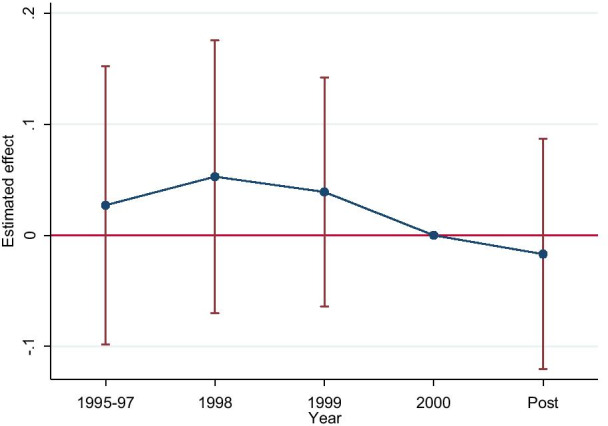
Event study, sales outcome, Canada and the US. *Notes*: Coefficients are displayed for 1995–1997, 1998 and 1999 (pre-policy) and for 2001–2005 (post- policy), relative to the excluded year of 2000 just prior to the year of policy implementation. Vertical bars around each point estimate represent the 95% confidence intervals

We also tested the validity of the pre-policy common trend assumption by conducting an event study for the sales outcome. This analysis re-estimated the DD regressions but replaced the event indicator with a series of indicator variables representing the years relative to the first post-GHW year. This event study allows a visual inspection of the parallel-trends assumption by examining any systematic differences in outcomes between Canada and the US in the pre-GHW period which could suggest non-parallel trends. Figure 4 shows that the coefficients for the pre-policy period indicators are not significantly different from zero, suggesting that there was  no difference in sales trends between Canada and the US prior to the GHWs.

##### Estimations of cluster robust errors in difference-in-differences analysis

Standard errors are often underestimated, and the null is overly rejected when the number of clusters (*G*) is small (especially when *G* = 2 in our case). Consequently, the fact that we did not find any significant effect even with these underestimated standard errors is reassuring and actually strengthens our null finding. However, we conducted additional estimation of standard errors using recently developed methods. Specifically, we used Young’s procedure [32], which has been shown to work well even for the case of G* = 1 [33]. We also used Pustejovsky and Tipton’s method [34], which corrected some issues with McCaffrey and Bell’s approach [35]. Applying these two procedures, we obtained *p*-values of 0.92 for Young’s procedure [32], and 0.54 for Pustejovsky and Tipton (2018)’s procedure [34]; each well exceeds the 10% significance level. These results confirm the robustness of our null findings.^10^

To summarize, we cannot find a specification that yields a credible role either to a dummy variable that shifts the intercept at the beginning of 2001, or to a dummy that impacts the slope of the trend in a significant and theoretically correct manner. Considered together, these regressions fail to provide evidence favoring the hypothesis that the introduction of GHWs in January 2001 reduced the level of sales. Nor do they indicate that there was an increase in the rate at which sales declined in periods following the introduction of GHWs.

## Incentive compatible messaging for 2022: a proposal

The failure of GHWs to detectably impact cigarette sales was influenced by three factors. One is that the messages were and are purely negative and they provided no direction for smokers to transition to a less risky mode of consumption. Second, the marketplace at that time had no risk-reduced close substitutes for cigarettes, whereas today it has. Third, in the current era all major global cigarette producers are supplying a menu of risk-reduced products and thus have less of an incentive to keep their cigarette purchasers hooked on one particular element in their product line.^11^

Psychologists have explored the role of stimuli in incentivising behavior changes. One summary of this work is Goldstein et al. [36], who explicitly include the case of health warnings:

For the most part, research has demonstrated that fear-arousing communications usually stimulate recipients to take action to reduce the threat. This general rule has one important exception, however: when the fear-producing message describes danger but the recipients are not told of clear, specific, effective means of reducing the danger, they may deal with the fear by ‘blocking out’ the message or denying that it applies to them. As a consequence, they may indeed be paralyzed into taking no action at all.

**Fig. 5:**
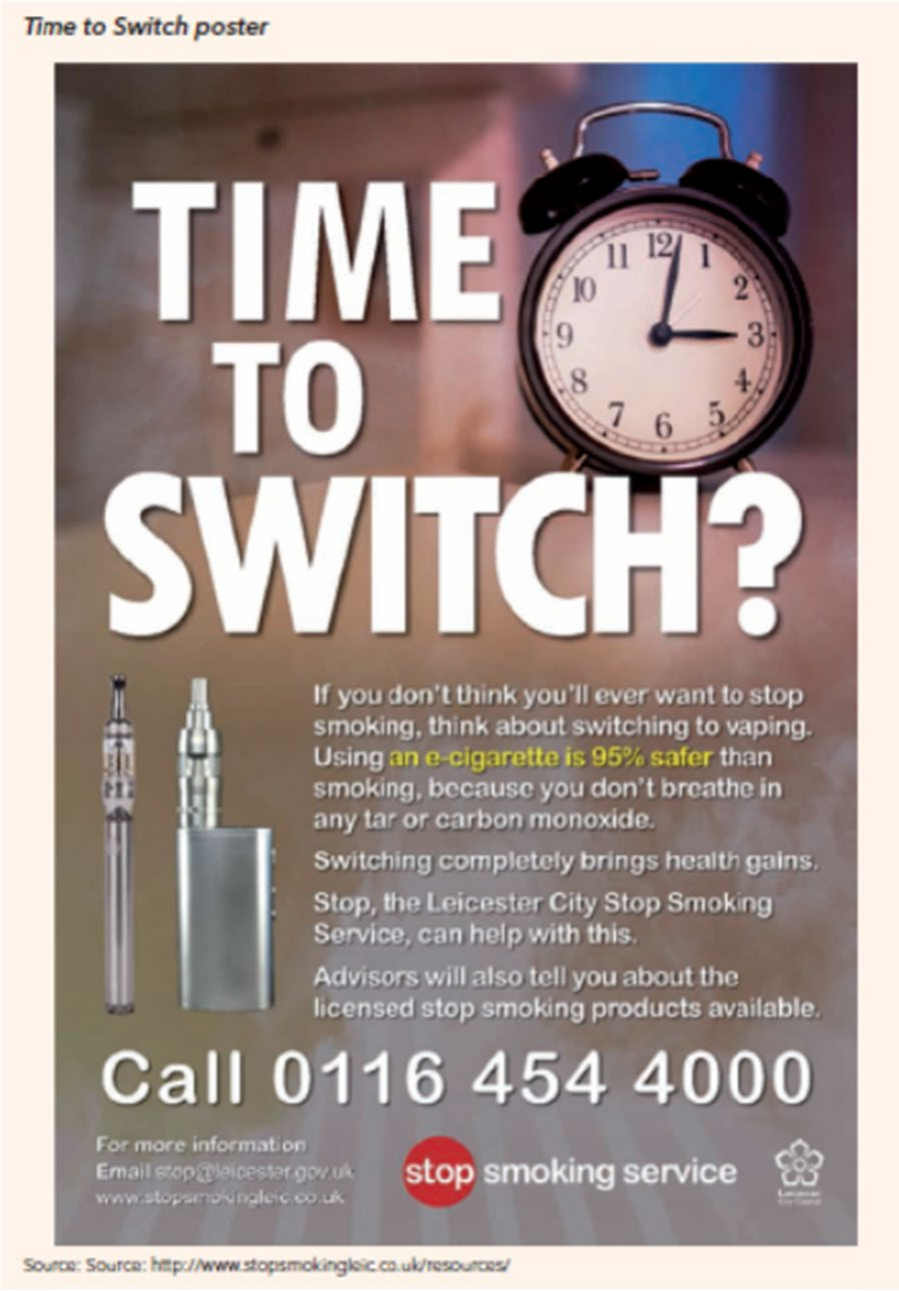
A positive ‘switch’ graphic adopted by City of Leeds, UK

Our proposal for a harm-reduction program is inspired by the City of Leicester, UK, program which implemented a graphic ‘switch’ campaign aimed at smokers in 2014 (see Fig. 5). In contrast to the GHWs promoted by the World Health Organization’s Framework Convention for Tobacco Control, Health Canada and the Food and Drug Administration, our proposal aims to transition smokers to a RRP rather than scare them into total nicotine abstinence.

We propose that, on one side of a cigarette pack, manufacturers be permitted to post a switch message that would direct smokers to a corporation’s own risk-reduced product line. Such messaging might direct users to a modern oral product, to an electronic cigarette or to a heated tobacco stick.

The new FDA regulations will require manufacturers to print a graphic on both the front and back of cigarette packs that occupies one half of the space. These graphic images are to be rotated from a supplied set of 13. Our proposal could take one of two forms: either producers would be permitted at the federal level to replace the mandated double negative warning with a combination of a negative and a positive (FDA-approved) message, or that individual states could petition for a positive message on one side rather than a negative message on each side. From an analytic standpoint, the latter would provide econometricians and biostatisticians with a quasi clinical trial and thus facilitate an ex-post examination of the efficacy of the positive/switch messaging. States that were early to legalize recreational marijuana might experiment.

This proposal is not without challenges. New product producers under current law must make a pre-market tobacco application (PMTA) to the FDA for product sale approval. In addition, in order to be able to claim a health ‘benefit’, producers must apply for a ruling that classifies the product as a modified risk tobacco product (MRTP). If the FDA approves the PMTA, and a MRTP request has been lodged, the FDA may rule (a) that the product involves reduced exposure to toxins, or (b) that, in addition to reduced exposure, the product is demonstrably less risky to health in the longer term. For example, the heat-not-burn product IQOS was ruled as falling under category (a) in July of 2020, but not under (b), because studies of potential longer-term risk impacts of IQOS use were not sufficiently convincing.

The approval process as of end-2020 appears to require years rather than months or weeks. The FDA has put in place very rigorous standards with the result that some manufacturers submit hundreds of thousands of pages of documentation in order to leave as little room for rejection as possible. This, in turn, demands many months of review by a team of FDA scientists. The number of applications by manufacturers to the FDA as of end-2020 is not publicly available.^12^ A solution to the timing issue would come in the form of a temporary fast-track categorization by the FDA. The FDA could issue a reduced exposure ruling (or not), within say one month of receiving a reduced-exposure application, that would be valid for one or two years in a limited number of experimenting states.

A key behavioral characteristic of such a program is that it is substantially incentive compatible. It would not have motivated or interested manufacturers when GHWs were introduced in Canada in 2001. Today, every major cigarette producer supplies risk-reduced tobacco or nicotine products to the market. Their websites universally indicate that their research and development expenditures are primarily directed to these products, and that they have invested billions in the process. Our proposal thus is incentive compatible for producers, because it encourages cigarette manufacturers to more vigorously promote a move towards a risk-reduced nicotine-delivery device in their own product range.

From the regulatory side, 2020 also differs from 2001. The new risk-reduced products currently on the market have gained the unequivocal initial support of Public Health England [37] and the Royal College of Physicians [38] as quitting devices for severely habituated smokers. Recent evidence indicates that vaping is a more successful route to quitting smoking than either patch therapy or nicotine gum treatment [39].

Corporate investment decisions are not attributable to philanthropy; producer websites do not all indicate that they wish to eradicate cigarettes, rather that they are committed to a wider range of products, with an emphasis on harm reduction. Profit reaped from selling risk-reduced products (including alcohol, gambling and certain drugs) is more socially tolerable than profit reaped from products that are cancer causing, that damage the heart and cause stroke. Society tolerates the generation of profit from the production and sale of alcohol, even though many thousands die each year in the US [40] from alcohol-related causes. Similarly, society tolerates the profit that results from both private and public gaming, even though the odds that the public faces in most state-owned lotteries are extraordinarily unfavorable. Thus, profits from risk-reduced nicotine products should, by some reasonable ethic, be viewed equally.

The corollary of permitting manufacturers to encourage switching to risk-reduced products via cigarette-pack messaging is that such promotion might be encouraged more widely. In particular, in jurisdictions that currently blank out cigarette displays in what were once ‘power walls’, but simultaneously permit the sale of alcohol in corner stores and gas stations, or in jurisdictions where lottery terminals are placed directly above the candy section at the check-out, society would have to confront the question of whether messaging that encourages a switch to risk-reduced nicotine products should be permitted, or even encouraged.

While our messaging proposal is specific, it has wider implications. Some nicotine markets have seen regulators treat e-cigarettes as being not very different from combustible tobacco products in that they levy similar excise taxes on each product. British Columbia, for example, imposes an excise rate on heat-not-burn products that is identical to the rate on cigarettes. Such policies are counter productive if harm reduction is the goal. Some 30 US states have now imposed taxes on e-cigarettes^13^, and the variation in such rates is enormous [41].^14^ In addition, there exists a high degree of ignorance on the part of the consuming and non-consuming public in relation to risk-reduced products. In the US, Dave et al [43] found that about one person in six correctly understands their nature, and that such ignorance grew during the late stages of the EVALI outbreak. EVALI is a misnomer for the condition that killed almost 70 users of illegal marijuana in the US during 2020. Technically, the term stands for ‘E-cigarette or Vaping Associated Lung Injury’. In fact, virtually all fatalities were found to involve the consumption of illegal cannabis liquids contaminated with vitamin E acetate. Similar rates of ignorance characterize the Canadian market [44]. Hence a more general information program is needed to counter misinformation.

Finally, media coverage of e-cigarettes has focused almost completely upon teen use, while neglecting the potential of ANDs to redirect adults who smoke to an alternative risk-reduced product. The positive switch messaging that we advocate targets only those who purchase cigarette packs, not under-age youth. Hence it provides no incentive for youth to vape.

In summary, risk-reduced products exist in many once smoking-only markets. Policies designed to reduce smoking further could adopt a harm-reduction approach through a judicious process that informs the public of the relative risks associated with consuming nicotine in different forms.^15^

## Conclusion

The relationship between health and smoking is unequivocal: smoking causes uncountable premature deaths each year throughout the world. Policies that reduce smoking are therefore valuable. But the interpretation of research results is frequently ideologically driven. In this paper, we have explored higher frequency data on cigarette sales, smoking prevalence and production in Canada, which have been long available but unused. No statistically significant evidence is contained in these data that suggests GHWs are effective in reducing combustible cigarette consumption. Conventional models grounded in prices and time trends, with no discernable breaks in the level or trend of the outcomes, drive the series. In addition to the results based upon monthly data, we have drawn attention to several drawbacks of annual data based upon surveys. That GHWs on cigarette packages might reduce smoking prevalence by almost 20%, as claimed by earlier researchers, represents an excess of enthusiasm over evidence.

In the United States, the FDA has labored for well over a decade for negative GHWs to appear on cigarette packs. Our econometric results indicate that we should not expect any notable decline in sales or consumption as a result of that measure. The main impact of GHWs will be to add to the anti-smoking culture that has grown steadily over several decades, and this may impact smoking in the longer term.

In a recent open letter to health researchers, the editors of eight economics journals specializing in health issued a statement in support of careful data-driven research that did not necessarily arrive at the ‘right’ conclusions [45]. This statement was motivated by a concern that too much research in the health area is driven by a zeal to uncover ‘good news’, and that negative or inconclusive evidence on specific policy measures or treatment measures attracts insufficient attention. Studies that show GHWs have an impact on quit intentions frequently avoid asking if those heightened intentions translate into observable abstinence from smoking.

Social scientists favor the provision of information that facilitates accurate decision making. But that information should be efficient: can the goal of smoking reduction be attained with more effective messaging, if commonly used negative messaging falls short? The current nicotine market encompasses numerous alternative nicotine delivery devices that are likely an order of magnitude less risky than combustible cigarettes. Messaging on cigarette packs that directs smokers to these products form positive signals that are at the same time compatible with the goals of nicotine suppliers—to earn revenue and profit. Research shows that e-cigarettes are an effective method of reducing smoking, primarily because they better mirror the smoking experience than alternative quitting methods.

## Data Availability

Data used in this study are available from Statistics Canada (CANSIM table 303-0062 and 303-0007) and the Canadian Tobacco Use Monitoring Surveys (CTUMS).

## Footnotes

1 It was scheduled to start in June 2021 but has been postponed to January 2022 due to COVID-19 [1].

2 These estimates have also been used by a notable set of researchers, to estimate benefits that would accrue to a GHW requirement on cigarette packages in the US [4]. A large literature on how to compute the costs and benefits of tobacco control regulations has developed recently. It focuses upon whether smoking initiation is a rational decision, and whether smokers know the real health cost of smoking [4–6]. This debate is reviewed in Levy et al. [7].

3 Chipty, in research commissioned by the Australian government claimed that plain packaging reduced smoking prevalence by one half of one percentage point [9]. This is the largest impact in the econometric literature, and itself has come under criticism from Viscusi [10] and Dryden [11].

4 Electronic cigarettes are the most popular form of ANDs. Heat-not-burn products (IQOS is the market leader) have made inroads particularly in Japan and Korea and are now available in North America. Modern oral tobacco, in the form of moist pasteurized tobacco or synthesized nicotine pouches (a.k.a. ‘snus’) are the third major component of the ANDs family.

5 Hammond [17] suggested that, because not every pack of cigarettes in the early part of 2001 carried the warnings, tests based on early-2001 data might not be fruitful: the warning labels were required to be on all packs printed from the start of 2001, and thus some inventory of packs without GHWs were in circulation early in the year. However, the CTUMS begin in the month of February each calendar year.

6 We as authors have used the data from both sets of surveys in earlier work. We have also replicated the results of HCF exactly, using the data posted as an on-line annex to their paper.

7 The smuggling took the form of domestically manufactured cigarettes being exported free of taxes and imported illegally. This smuggling accounted for perhaps one third of the cigarette market in some provinces in the early nineties [24]. If we were to include the smuggling period in our analysis the price index for cigarettes would be subject to error, because it is based only upon the higher priced legal product.

8 These results are available from authors upon request.

9 Canadian cigarette taxes are composed of four elements: federal excises (specific), provincial excises (specific), federal sales tax (uniform across-provinces ad valorem), and provincial (ad valorem) sales tax of varying rates (sometimes zero). The sales taxes account for a small fraction of total tax revenues generated. Since the ad valorem taxes depend upon pricing by the manufacturers, the tax wedge is endogenous to the process—in contrast to the excise components. Hence, in the tax variable we use only the exogenous excise components. The economy-wide index is aggregated from the month-by-month provincial excise rates. We used provincial populations as weights, rather than quantity of cigarettes, to avoid endogeneity.

10 These results are available from authors upon request.

11 We are not claiming that profitability on all products is the same at the present time. Traditional cigarettes benefit from scale economies and a century of automated production. But we can see no reason why the production of a heat-stick, for example, should be more costly than a cigarette once scale has been achieved. We are grateful to David Sweanor for pointing us to the profitability issue. He also guided us to the Goldstein et al. reference in the following paragraph.

12 Small manufacturers of new products cannot afford to underwrite applications having the scope of those submitted by global corporations, and this hinders both the introduction of new risk-reduced products and competition in the marketplace.

13 The US also increased the minimum legal age of purchase for all tobacco products to 21 as of December 2019.

14 An emerging literature evaluates effects of e-cigarette taxes in the US [13,42].

15 We have analyzed data detailing cigarette shipments from bond for both the US and Canada for the period 2011–2019. Significant declines are evident for Canada since late 2018, and for the US since 2017. In a separate note we illustrate that these declines cannot be explained by traditional factors such as price and trend. They are attributable to the arrival of a disruptive technology in the tobacco market—alternative nicotine delivery devices.
